# Proteomics Analysis of Porcine Endometrial Cell-Derived Extracellular Vesicles Involved in Embryo Attachment

**DOI:** 10.1016/j.mcpro.2025.100942

**Published:** 2025-03-11

**Authors:** Seonggyu Bang, Ahmad Yar Qamar, Sang-Yeop Lee, Ayeong Han, Heejae Kang, Bereket Molla Tanga, Sung Ho Yun, Hye Sun Park, Seung Il Kim, Won Gi Yoo, Islam M. Saadeldin, Sanghoon Lee, Jongki Cho

**Affiliations:** 1 College of Veterinary Medicine and Research Institute for Veterinary Science, Seoul National University, Seoul, Republic of Korea; 2 College of Veterinary Medicine, Chungnam National University, Daejeon, Republic of Korea; 3 College of Veterinary and Animal Sciences, Jhang, Sub-Campus of University of Veterinary and Animal Sciences, Lahore, Pakistan; 4 Korea Basic Science Institute (KBSI), Chungcheongbuk-do, Republic of Korea; 5 Comparative Medicine Department, King Faisal Specialist Hospital and Research Centre, Riyadh, Saudi Arabia

**Keywords:** endometrium, extracellular vesicle, proteomics

## Abstract

Maternal–embryo interactions play a critical role in early mammalian development, with extracellular vesicles (EVs) playing a key role in intercellular communication. Recent studies have focused on the mechanisms by which maternal-derived factors, such as RNA, proteins, and metabolites influence gap junctions, EVs, and direct cell-to-cell interactions, contributing to embryonic development. In this study, using a proteomics approach, we investigated the impact of EVs secreted from porcine endometrial cells (pEECs) and their protein cargoes on embryonic development. We characterized EVs isolated from pEECs (pEEC-EVs) during the diestrus stage using a nanoparticle tracking analysis and cryo-transmission electron microscopy. Furthermore, the effects of pEEC-EVs with or without hormone treatment on the *in vitro* attachment of hatched blastocysts were evaluated. The attachment rate of porcine embryos was significantly higher for pEEC-EVs in the hormone treatment group than the control group (23.0 ± 1.7% *versus* 36.9 ± 1.9% for control and pEEC-EVs, respectively). Furthermore, hormone treatment altered the expression of proteins involved in cellular organization, protein transport, and immunity. Proteomic analysis revealed distinct biological processes between groups: control EVs supported cytoskeletal organization and adhesion, while hormone-treated EVs were enriched in protein transport, immune regulation, and stress response pathways. Key signaling pathways, including VEGFA-VEGFR2, focal adhesion, and TGF-β, were modulated, influencing implantation and embryogenesis. EVs play a crucial role in maternal–embryo interactions, optimizing implantation conditions and supporting embryo-derived stem cell establishment. These findings enhance our understanding of EV-mediated communication and suggest potential applications for improving reproductive health and assisted reproductive technologies.

## Highlights

• Isolation of extracellular vesicles derived from porcine endometrial cells.
• Effects of endometrial-derived extracellular vesicles (evs) on embryo attachment.
• Proteomic analysis of endometrial-derived extracellular vesicles.

## Graphical Abstract

**Figure:**
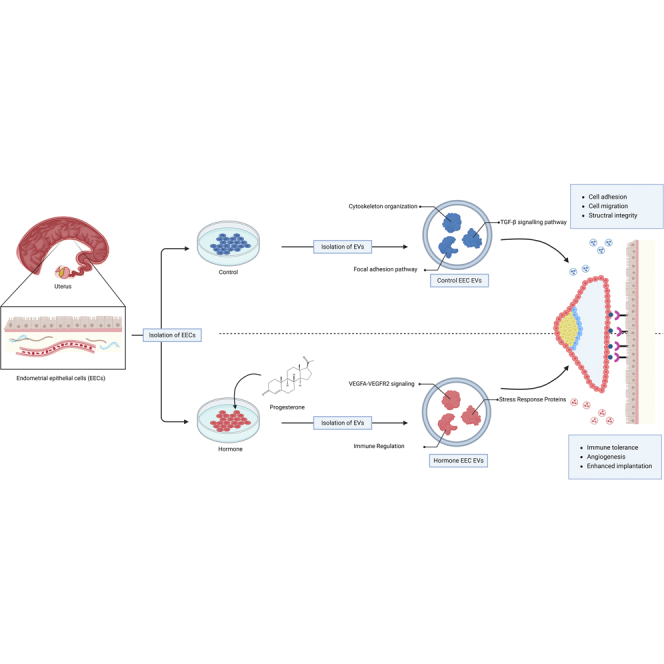

## In Brief

This study examines extracellular vesicles from porcine endometrial cells and their role in embryo attachment under hormonal influence. Proteomic analysis identified differentially expressed proteins linked to cellular organization, immunity, and protein transport. Key pathways, including VEGFA-VEGFR2, focal adhesion, and TGF-β, were associated with implantation. Findings suggest porcine endometrial cell–derived extracellular vesicles modulate the uterine microenvironment, enhancing embryo implantation and advancing reproductive technologies.

Maternal–embryo crosstalk, a dynamic interplay of molecular signals and cellular interactions, plays a pivotal role in shaping early embryonic development. This intricate dialog begins even before fertilization, with maternal factors present in the oocyte and surrounding cumulus cells influencing sperm selection, gamete fusion, and subsequent embryonic development (1). Following fertilization, the maternal environment continues to exert profound influences on embryonic gene expression, epigenetic modifications, and metabolic programming (2, 3). Maternal-derived factors, including RNAs, proteins, and metabolites, are transported to the developing embryo *via* various mechanisms, such as gap junctions, exosomes, and direct cell-cell interactions, modulating key developmental processes (4, 5). Conversely, the developing embryo secretes signals that orchestrate maternal physiological adaptations necessary for successful implantation, placental development, and maintenance of pregnancy (6, 7, 8). This bidirectional communication is critical for maintaining the conditions necessary for embryonic growth and development (9, 10). The disruption of maternal–embryo crosstalk can have severe consequences, including implantation failure, early pregnancy loss, developmental abnormalities, and fetal growth restriction (11). Thus, understanding the molecular mechanisms underlying this interaction is crucial not only for deciphering the complexities of early mammalian development but also for improving assisted reproductive technologies and pregnancy outcomes in both human and animal models (12, 13).

Intracellular communication is vital during embryogenesis, as it regulates and coordinates various processes necessary for forming a complex multicellular organism (14, 15). Precise spatial and temporal regulation of intracellular signaling pathways is crucial for cell proliferation, differentiation, migration, and apoptosis, ultimately leading to proper tissue and organ formation (15).

Extracellular vesicles (EVs) have emerged as critical mediators of intercellular communication in the context of embryonic development (16, 17). EVs are secreted by various cell types and carry diverse cargo, such as proteins, lipids, RNAs, and microRNAs. By facilitating the transfer of these bioactive molecules, EVs influence numerous developmental processes, including cell proliferation, differentiation, and migration (18).

Endometrial cell–derived EVs carry factors that modulate trophoblast cell behavior, enhance adhesion, and remodel the extracellular matrix, thereby contributing to embryo implantation (19, 20). These EVs are essential for creating a supportive environment for the embryo, ensuring proper communication between the endometrium and the developing embryo (21).

Proteins contained within EVs have important functions in embryonic development (9, 22). Proteomics, a powerful analytical tool, has been used to evaluate the biological significance of proteins in EVs and potential applications in reproductive medicine (23, 24). The identification of specific proteins associated with successful implantation and embryonic development can provide a basis for the development of targeted interventions to enhance reproductive outcomes and improve assisted reproductive technologies (25).

In this study, we analyzed the impact of hormonal treatment on proteins in porcine endometrial cell–derived EVs (pEEC-EVs) using proteomics to assess their influence on embryonic development. Our findings could serve as a cornerstone for applying EV-mediated microenvironmental modulation in the development of *in vitro* embryo production and implantation models.

## Experimental Procedures

### Ethical Statment

Ethical approval was not required for this study because the samples were obtained from pigs and cattle slaughtered at a commercial abattoir. The animals were not specifically slaughtered for research purposes, and sample collection was performed postmortem during routine slaughter processes.

### Chemicals

Unless otherwise specified, all chemicals and reagents were purchased from Sigma-Aldrich.

### Preparation of pEEC-EVs

#### Primary Cell Culture of pEECs

Pig uterus was obtained from a nearby slaughterhouse. Uterus from the luteal phase was used. The uterus was opened to collect the uterine tissue using sterile surgical scissors. The collected uterus was washed with PBS containing 1% penicillin-streptomycin, after which the endometrial tissue was harvested using a sterile scalpel. The harvested tissue was then minced in 0.25% trypsin-EDTA. The minced tissue was incubated at 38 °C for 5 min for dissociation into single cells. After incubation, the tissue was washed with PBS containing 1% penicillin-streptomycin. Finally, the cells were cultured in Dulbecco’s modified Eagle’s medium (DMEM) supplemented with 10% fetal bovine serum (FBS). The cells were passaged after reached over 80% confluence to maintain the cell culture. Cells were cultured in DMEM without FBS, and the medium was collected for EV isolation. To analyze the effects of pEEC-derived EVs on embryo implantation and the contributing factors, we conducted experiments with two groups: a control group and a hormone-treated group. Since the implantation period occurs in the late diestrus phase, we extracted EVs under conditions that mimicked the diestrus state, during which progesterone is dominant in the uterus. The hormone treatment was based on previous studies; we obtained the conditioned medium by treating 2 to 3 passage cells with hormone concentrations mimicking porcine blood hormone levels (10 pg/ml E_2_ + 35 ng/ml P_4_) (26).

#### Isolation of EVs

EV extraction was performed following our previously described protocol (27). The conditioned medium derived from pEECs underwent sequential centrifugation at progressively higher speeds, 400*g* for 10 min, 2000*g* for 30 min, and 10,000*g* for 60 min, to eliminate apoptotic bodies and cellular debris. The resulting supernatant was filtered using a 0.20 μm syringe filter, and the EV pellet was subsequently isolated through ultracentrifugation at 100,000*g* for 180 min.

### Nanoparticle Tracking Analysis

Nanoparticle tracking analysis (NTA) is a technique used to determine the size, concentration, and distribution of nanoparticles in a liquid suspension by tracking their motion under laser illumination. An NTA was performed using the NanoSight NS300 (Malvern Panalytical). EV samples were diluted in PBS to a final volume of 1 ml for analyses. In accordance with the manufacturer's guidelines (NanoSight NS300 User Manual, MAN0541-01-EN-00, 2017), the particle concentration was adjusted to achieve an optimal measurement range of 20 to 100 particles per frame. The characteristics of EVs in each group were determined, including the particle size distribution, mean size, and concentration differences.

### Cryo-Transmission Electron Microscope

Cryo-transmission electron microscope (Cryo-TEM) is an advanced imaging technique that allows the visualization of biological nanoparticles, such as EVs, in their near-native hydrated state by rapidly freezing samples in vitreous ice. As in our previous study, cryo-TEM was employed to visualize EVs (28). Specifically, 3.5 μl of the eluted EVs was placed onto glow-discharged Quantifoil R1.2/1.3 Cu 300 grids (Quantifoil). The grids were then rapidly vitrified in liquid ethane using a Vitrobot Mark IV (Thermo Fisher Scientific). The Vitrobot chamber was maintained at 100% humidity and 4 °C, with a blotting time of 5 s. Cryo-TEM images were captured using a Glacios microscope (Thermo Fisher Scientific) operating at 200 kV, equipped with a 70-μm C2 aperture and set to a magnification of 730,00×. Image acquisition was performed using a Falcon III direct electron detector (Thermo Fisher Scientific) in linear mode, utilizing a 100-μm objective aperture. The protein cargo of EVs was further examined *via* proteomic analysis, as detailed below.

### Effects of pEEC-EVs on Porcine Embryonic Attachment

#### Preparation of Embryos

We followed the *in vitro* maturation (IVM) method used in our previous study (29, 30). Briefly, porcine ovaries were obtained from a nearby slaughterhouse and transported to the laboratory at 34 to 36 °C in 0.9% saline solution containing 75 mg/ml penicillin and 50 mg/ml streptomycin. Cumulus–oocyte complexes (COCs) from porcine antral follicles (3–8 mm in diameter) were aspirated using a 10 ml syringe and an 18-gauge needle. To separate the sediments, the aspirated follicular fluid was placed in a 15 ml conical tube and kept at room temperature for 5 min. COCs of high quality were chosen and washed three times in Hepes-buffered Tyrode's medium (TLH) containing 0.05% (w/v) polyvinyl alcohol (TLH-PVA). IVM of the selected COCs was performed using bicarbonate-buffered tissue culture medium 199 (TCM-199, Gibco, BRL) supplemented with 10% (v/v) porcine follicular fluid, 0.91 mM sodium pyruvate, 0.57 mM l-cysteine, 10 ng/ml epidermal growth factor, 1 μg/ml insulin, 10 IU/ml human chorionic gonadotropin, 10 IU/ml equine chorionic gonadotropin, and 75 μg/ml kanamycin sulfate for 22 h at 38.5 °C in an environment of 5% CO_2_. Following the initial incubation of 22 h, COCs were incubated for an additional 22 h at 38.5 °C and 5% CO_2_ using a hormone-free IVM medium.

Briefly, following 44 h of IVM, the removal of cumulus cells from matured COCs was performed by gentle pipetting in an IVM medium supplemented with 0.1% hyaluronidase. Denuded oocytes were then treated with an activation solution (280 mM mannitol solution containing 0.05 mM MgCl_2_ and 0.01 mM CaCl_2_) and subjected to an electric pulse using two direct currents at 120 V/cm for 30 μs using the Electro-cell Manipulator 2001 (BTX) to produce parthenogenetic embryos. The activated oocytes were cleaned with porcine zygote medium-5 (PZM5; IFP) and were then added to 25 μl of PZM-5 coated with mineral oil on a Petri plate. Finally, activated oocytes were cultured in a humidified environment of 5% O_2_, 5% CO_2_, and 90% N_2_ at 38.5 °C. Hatched blastocysts were cocultured with feeder cells on day 7 of embryo culture. The coculture was conducted independently seven times, with 81 hatched blastocysts used per group. Additionally, during the coculture of embryos and feeder cells, pEEC-derived EVs were administered at a concentration of 1.5 × 10^7^ particles/ml, following our previous study (29, 31).

#### Preparation of Feeder Cells

Mouse embryonic fibroblasts (MEFs) were used as feeder cells; the cell lines and methods are described in our previous study (32). The cryopreserved cells were thawed in a 38 °C water bath and then washed three times with PBS at 1500 rpm for 3 min each. MEFs were maintained in DMEM (Gibco), supplemented with 10% fetal bovine serum, 1% nonessential amino acids (Gibco), 1% GlutaMAX (Gibco), 0.1 mM β-mercaptoethanol, and 1% antibiotic-antimycotic (Gibco). The cultured cells were incubated at 37 °C under 5% CO_2_ in a humidified environment. To render ICR MEF feeder cells mitotically inactive, the cells were treated with mitomycin C (10 μg/ml; Roche) for 2 to 2.5 h. These treated MEF feeder cells were then seeded at a concentration of 5 × 10^5^ cells/ml in a 6-well plate coated with EmbryoMax 0.1% Gelatin Solution (Millipore) and supplemented with 2 ml of MEF culture medium. Passage 3 to 4 cells were utilized to support the growth of blastocysts.

### Liquid Chromatography-Mass Spectrometry

Tryptic peptide samples were prepared through in-gel tryptic digestion following protein separation *via* 12.5% SDS-PAGE. Chemical contaminants were removed, and peptides were enriched using a trapping column (75 μm inner diameter, packed with 5 μm C18 particles, Acclaim PepMap100, Thermo Fisher Scientific). Enriched peptide solutions were subsequently concentrated and separated using reversed-phase liquid chromatography on an Ultimate 3000 RSLC nano system (Thermo Fisher Scientific) with a binary solvent system composed of 0.1% formic acid (solvent A) and 80% acetonitrile in 0.1% formic acid (solvent B). Chromatographic separation was performed on a 15-cm analytical column packed with 2 μm C18 particles (Acclaim PepMap RSLC, Thermo Fisher Scientific) using a linear gradient of solvent B from 5% to 95% at a flow rate of 300 nl/min over 100 min. Peptides were analyzed using a Q Exactive Plus mass spectrometer (Thermo Fisher Scientific) in data-dependent acquisition mode. Each full MS scan (m/z range: 300–2000) was followed by 3 MS/MS scans targeting the most abundant precursor ions with dynamic exclusion settings. MS/MS analysis was performed in triplicate for each sample to ensure data robustness. Mass spectrometric raw data were processed using MASCOT, version 2.4 (Matrix Science). Database searches were conducted against the UniProt Sus Scrofa proteome database (UP000008227_9823) with the following parameters: enzyme specificity set to trypsin/P; missed cleavages, two; peptide mass tolerance, ±0.8 Da; peptide fragment tolerance, ±0.8 Da; peptide charge, 2+, 3+, and 4+; static modifications, carbamidomethyl; and dynamic modification, oxidation (Met). The data were filtered using a false discovery rate (FDR) threshold of <1% at both peptide and protein levels. Protein quantification was performed using the mol% calculated from the emPAI value. For further comparative and functional analyses, proteins with expression levels below 0.01 mol% and ribosomal protein L were excluded. Next, to distinguish differentially expressed proteins (DEPs), a cut-off value of −0.5 < log_2_(hormone/control) < 0.5 was applied.

### Protein Analysis

To analyze the DEPs, DAVID (Database for Annotation, Visualization, and Integrated Discovery), WikiPathways, MSigDB (Molecular Signatures Database), and STRING (functional protein association networks) were used. Each tool was employed to identify biological pathways and functional networks associated with the DEPs. Using DAVID, Gene Ontology terms and KEGG pathways were examined. WikiPathways was used to explore additional biological pathways. MSigDB was utilized to investigate connections to signaling pathways and physiological processes (33).

### Statistical Analysis

Statistical analyses were performed using SPSS version 26 (IBM Corp., Armonk). To compare parameters between the two groups, independent two-sample *t*-tests were performed. A *p*-value of less than 0.05 was considered statistically significant for all comparisons.

### Statistical Analysis Experimental Design & Statistical Rationale

The proteomic analysis was conducted using three biological replicates per group to ensure reproducibility and account for biological variability. Each biological replicate was derived from independent endometrial cell cultures under identical conditions. Technical replicates were performed in triplicate to increase the robustness of protein quantification and reduce measurement errors.

An FDR threshold of <1% was set at both peptide and protein levels to control for false-positive identifications. Statistical comparisons were performed using independent two-sample t-tests, with a *p*-value of <0.05 considered statistically significant.

This experimental design ensures that our results are statistically robust, allowing for reliable identification and quantification of DEPs between control and hormone-treated EVs.

## Results

### Characterization of pEEC-EVs

**Fig. 1:**
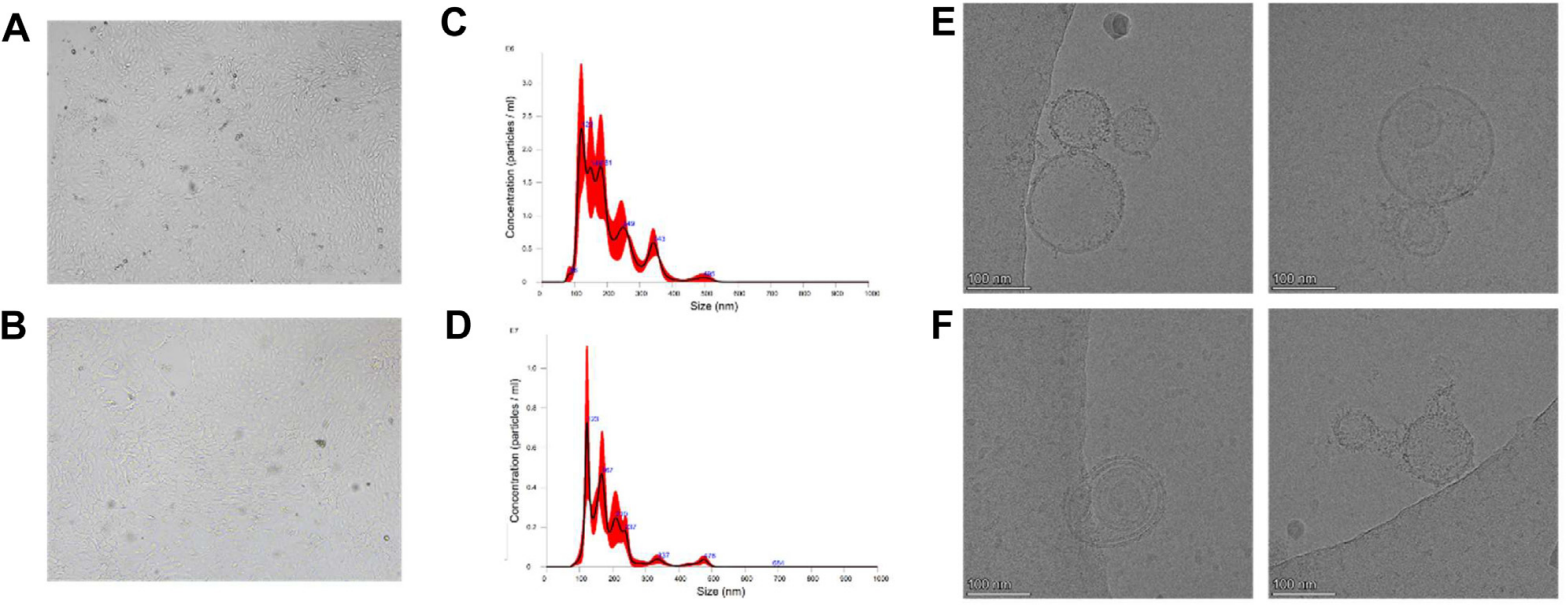
**Microscopic images of cultured control pEECs and hormone-treated pEECs**. *A* and *B*, size distribution plot, as per nanoparticle tracking analysis. *C*, EVs derived from porcine endometrial cells; control. *D*, EVs derived from hormone-treated porcine endometrial cells. *E* and *F*, cryo-TEM images of extracellular vesicles (EVs) derived from control and hormone-treated porcine endometrial epithelial cells (pEECs). The scale bar in (*E* and *F*) represents 100 nm.

Potential contaminants such as protein aggregates and cellular debris were minimized through rigorous centrifugation and filtration steps. Purity was confirmed by the absence of larger particles (>200 nm) in NTA and Cryo-TEM images, with minimal co-isolated proteins detected in proteomic analyses. pEEC-EVs were visualized and characterized. The NTA results showed that the control group had a size of 201.9 ± 18.8 nm, while the hormone group had a size of 188.0 ± 13.2 nm. In addition, the morphology of the isolated EVs was visualized through cryo-TEM (Fig. 1).

### Embryonic Outgrowth

**Fig. 2:**
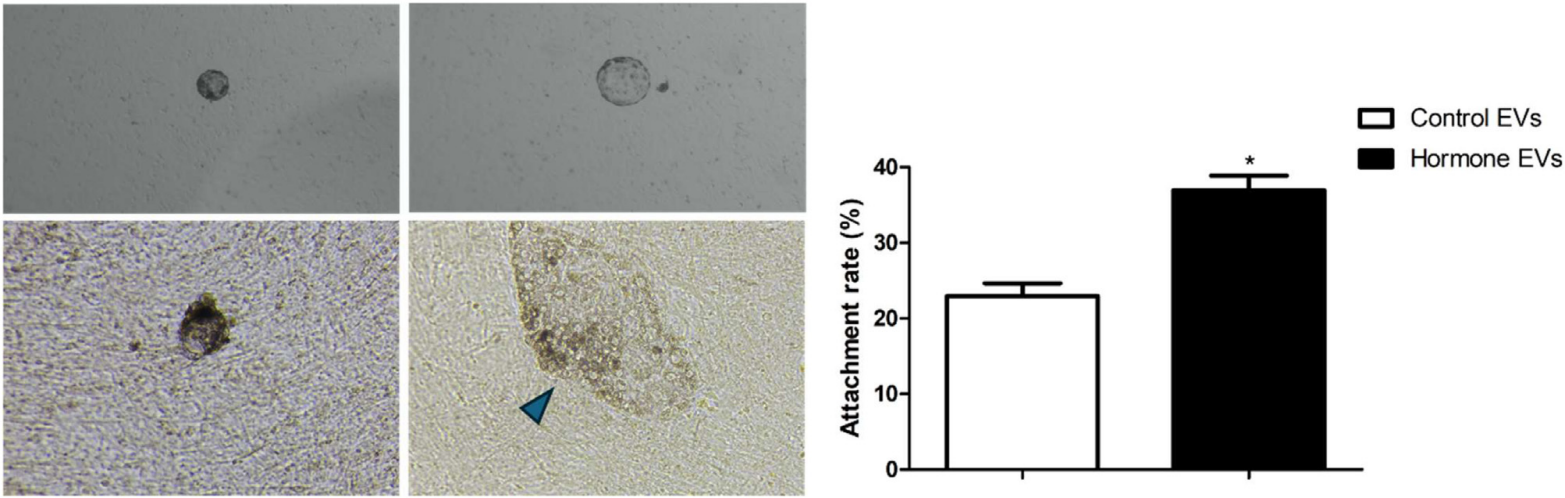
**Effects of EEC EVs on embryonic attachment.** Morphology of ES like cell and, bar graph for embryonic attachment.

Attachment of porcine-hatched blastocysts was induced using ICR MEFs as feeder cells. We attempted to mimic the maternal microenvironment at implantation by adding pEEC-EVs to the experimental group. We observed a significantly higher attachment rate of porcine embryos on pEECs in the treatment group than in the control group (Fig. 2).

### Signaling Pathways Related to Implantation in pEEC EVs

#### Detection of Proteins

**Fig. 3:**
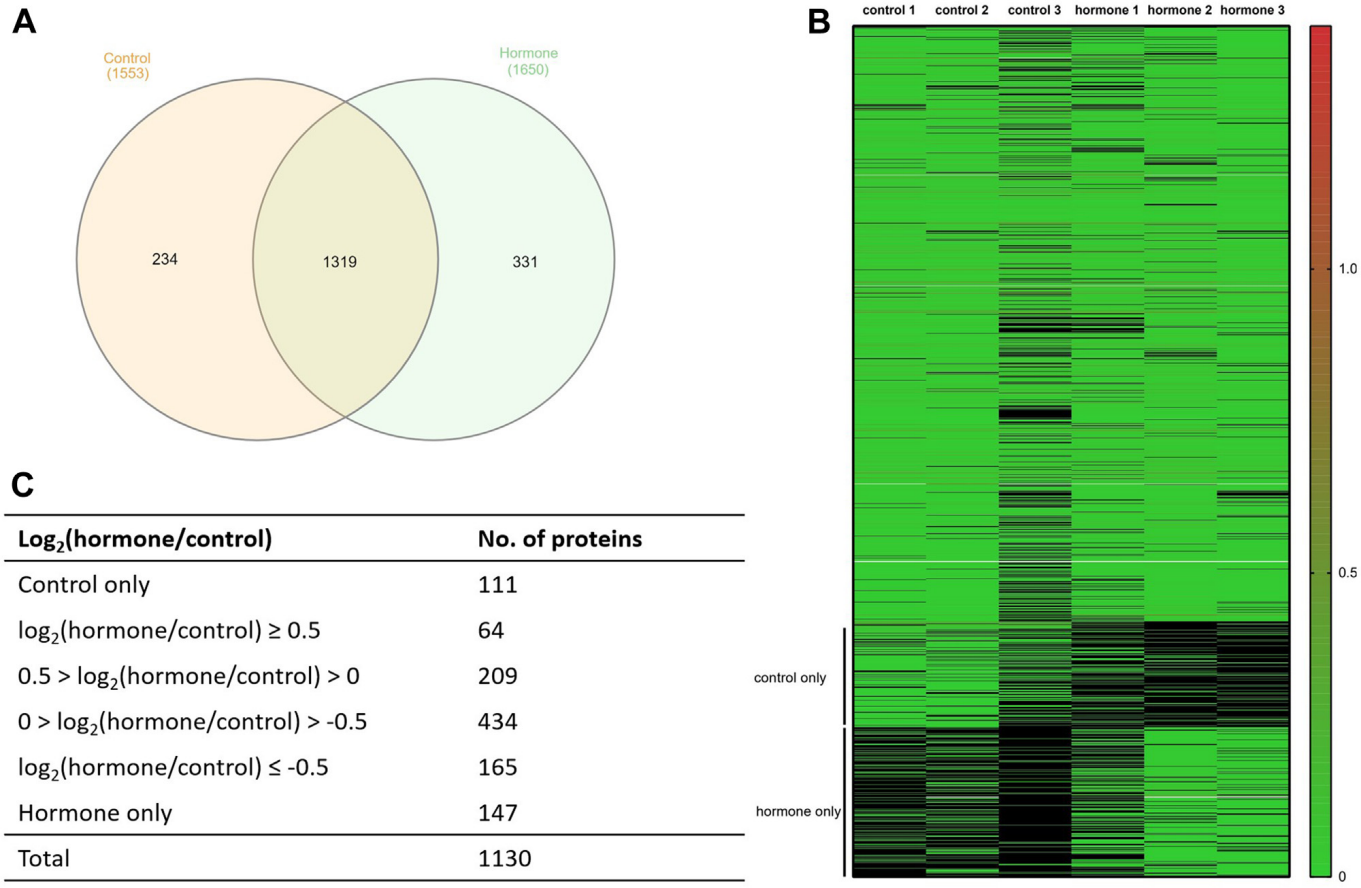
**Identification and quantitative evaluation of the proteome of exosomes derived from normal and hormone-treated endometrial cells.***A*, a Venn diagram visualizing the number of identified proteins from exosomes derived from normal and hormone-treated porcine endometrial cells. *B*, a heatmap evaluating the similarity between the normal and hormone-treated groups used in the experiment. *C*, the selection criteria and number of proteins for functional assessment. The mol% ratio values of the detected proteins were converted to log2 for comparison, and DEPs were determined based on the thresholds of 0.5 and −0.5.

A total of 1884 proteins were detected based on an FDR value of less than 1 across the two samples. In the control group, 1553 proteins were identified, 1650 proteins were identified in the hormone treatment group, and 1319 proteins were detected in both groups (Fig. 3*A*). Each sample was analyzed in triplicate, and proteins expressed in at least two replicates were compared. The similarity of the total secreted proteins was visualized using a heatmap (Fig. 3*B*). Ultimately, 487 proteins were retained, including 147 proteins detected only in the control group, 111 proteins only in the hormone group, and 229 proteins commonly expressed in both groups (Fig. 3*C*).

### Gene Ontology Analysis: Biological Processes

**Fig. 4:**
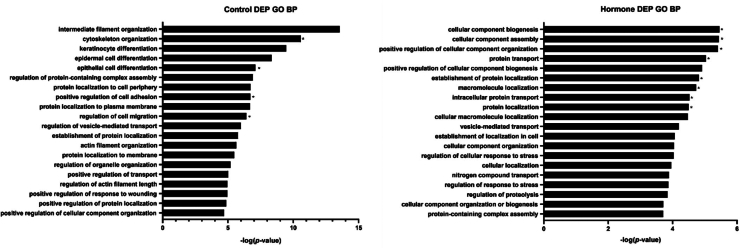
**The gene ontology results of DEPs identified in the control and hormone-treated groups.** The top 20 biological processes (BP) of the detected proteins in both groups were identified based on the *p*-value. An *asterisk* (∗) indicates the biological processes likely related to embryonic development and implantation.

The top 20 biological processes from the DAVID Gene Ontology analysis for the two groups were compared based on -log2 (*p*-values) (Fig. 4). In the control group, the biological processes related to embryonic development and implantation were obtained, including cytoskeleton organization, epithelial cell differentiation, positive regulation of cell adhesion, and regulation of cell migration. In the hormone treatment group, various processes, such as cellular component biogenesis, cellular component assembly, positive regulation of cellular component organization, protein transport, establishment of protein localization, macromolecule localization, intracellular protein transport, and protein localization, were identified.

In both groups, most DEPs were related to protein localization and transport, which are essential for cellular function and signal transduction in embryos. In particular, DEPs in the control group were related to protein localization in the plasma membrane, cytoplasmic membrane, and pericellular region, while DEPs in the hormone treatment group were related to protein transport, intracellular protein transport, and intracellular protein localization. Cellular component organization appeared to play a significant role in both groups. DEPs in the control group were related to cytoskeleton organization and organ organization, while those in the hormone group were involved in cellular component biogenesis and assembly.

DEPs in the control group were mainly involved in cytoskeleton organization and cell movement, indicating enhanced embryo mobility and favorable conditions for implantation. In contrast, DEPs in the hormone treatment group were mainly involved in processes related to the biogenesis and assembly of cellular components, focusing on the active production and organization of new cellular components. Processes related to the regulation of the stress response were also identified, indicating the importance of biological processes advantageous for responding to environmental changes.

### DEP Analysis Using Wikipathway

We analyzed the key signaling pathways of endometrial-derived EVs in the control and hormone-treated groups. In the control group, several significant pathways were identified (Table 1), including the VEGFA VEGFR2 signaling pathway (WP3888), which is crucial for angiogenesis and vascular permeability, supporting the endometrial microenvironment. The focal adhesion pathway (WP306) was also identified, emphasizing the importance of cell–matrix interactions for adhesion and migration.

**Table 1:** Top 10 enriched WikiPathways based on differentially expressed proteins in pEECs EVs

Group	Name	*p*-value
Control	VEGFA VEGFR2 signaling WP3888	1.513e-11
	Focal adhesion WP306	3.637e-09
	Complement system WP2806	3.822e-08
	Proteasome degradation WP183	4.919e-08
	Complement and coagulation cascades WP558	3.285e-07
	Regulation of actin cytoskeleton WP51	9.073e-07
	Pathogenic *Escherichia coli* infection WP2272	2.492e-06
	Common pathways underlying drug addiction WP2636	3.543e-06
	IL 24 signaling pathway WP5413	5.356e-06
	TROP2 regulatory signaling WP5300	6.770e-06
Hormone	VEGFA VEGFR2 signaling WP3888	1.839e-10
	Proteasome degradation WP183	7.663e-10
	Complement and coagulation cascades WP558	6.523e-08
	Complement system WP2806	1.149e-07
	IL 24 signaling pathway WP5413	1.830e-07
	Focal adhesion WP306	5.487e-07
	Complement system in neuronal development and plasticity WP5090	2.837e-06
	Parkin ubiquitin proteasomal system pathway WP2359	4.366e-06
	Common pathways underlying drug addiction WP2636	6.870e-06
	Alzheimer 39 S disease and miRNA effects WP2059	1.732e-05

Additionally, the complement system pathway (WP2806) was identified, suggesting the involvement of DEPs in immune responses, while the detection of the proteasome degradation pathway (WP183) highlights the role of protein degradation in cellular regulation. The complement and coagulation cascades (WP558) pathway points to interactions between immune responses and coagulation processes, and the regulation of the actin cytoskeleton (WP51) pathway underscores the role of cytoskeletal dynamics. Lastly, the pathogenic *Escherichia coli* infection pathway (WP2272) was identified, indicating the role of factors involved in the response to infections.

In the hormone-treated group, similar findings were obtained, including the identification of the VEGFA VEGFR2 signaling pathway (WP3888) and proteasome degradation pathway (WP183). The complement and coagulation cascades (WP558) and complement system (WP2806) pathways were also obtained, supporting the roles of DEPs in the immune response. The detection of the IL-24 signaling pathway (WP5413) suggests that DEPs were involved in cellular signaling and immune regulation.

Additional pathways, including focal adhesion (WP306) and complement system in neuronal development and plasticity (WP5090), indicate implications for adhesion and neuronal processes. The parkin ubiquitin proteasomal system pathway (WP2359) and Alzheimer’s disease and miRNA effects (WP2059) reveal potential changes in neurobiological and metabolic processes in response to hormonal treatment. Overall, these findings improve our understanding of the biological roles and signaling mechanisms of endometrial EVs in response to hormonal modulation.

### Expression Analysis of Proteins in Each Group Using WikiPathways

The proteins specific to each group were analyzed using WikiPathways. The analysis included 111 proteins expressed only in the control group and 147 proteins specifically expressed in the hormone-treated group. The major pathways identified in the control group, based on proteins uniquely expressed in this group and ranked by *p*-values, included TGF-β signaling in thyroid cells for epithelial-mesenchymal transition (WP3859), VEGFA VEGFR2 signaling (WP3888), focal adhesion (WP306), complement system (WP2806), and TGF beta receptor signaling (WP560). TGF-β signaling induces the epithelial-mesenchymal transition (EMT), which may enhance cell motility and adhesion, thereby contributing to the attachment of embryos to the uterine endometrium. The VEGF pathway plays a critical role in angiogenesis and cell growth, facilitating nutrient and oxygen supply during early embryonic development. Additionally, the focal adhesion pathway provides the structural basis necessary for stable attachment of the embryo to the uterine endometrium. The complement system and TGF-β receptor signaling also significantly impact immune responses and cellular growth and differentiation, playing vital roles in embryonic development.

In the hormone-treated group, the top five pathways associated with proteins uniquely expressed in this group, ranked based on *p*-values, were VEGFA VEGFR2 signaling (WP3888), proteasome degradation (WP183), complement and coagulation cascades (WP558), dengue 2 interactions with complement and coagulation cascades (WP3896), and parkin ubiquitin proteasomal system pathway (WP2359). The VEGFA VEGFR2 signaling pathway contributes to the nutritional support of the embryo, while the proteasome degradation pathway plays a crucial role in maintaining intracellular protein homeostasis by eliminating abnormal proteins. The complement and coagulation cascades pathway may also influence immune responses and, consequently, embryonic development. Furthermore, the parkin ubiquitin proteasomal system pathway is expected to contribute to the regulation of physiological functions and normal embryonic development through protein modulation (Table 2).

**Table 2:** Top 5 WikiPathways for proteins expressed exclusively in each group

Group	Name	*p*-value
Control	TGF beta in thyroid cells for epithelial mesenchymal transition WP3859	1.682e-06
	VEGFA VEGFR2 signaling WP3888	2.973e-06
	Focal adhesion WP306	1.094e-05
	Complement system WP2806	1.509e-04
	TGF beta receptor signaling WP560	1.923e-04
Hormone	VEGFA VEGFR2 signaling WP3888	4.436e-05
	Proteasome degradation WP183	6.862e-05
	Complement and coagulation cascades WP558	6.897e-04
	Dengue 2 interactions with complement and coagulation cascades WP3896	7.360e-04
	Parkin ubiquitin proteasomal system pathway WP2359	1.474e-03

These findings provide essential groundwork for elucidating the impact of EVs derived from endometrial cells on embryonic development and attachment.

### Molecular Signatures Database

The regulatory effects of endometrial-derived EVs were evaluated through a gene set enrichment analysis. In the control group, the following pathways were identified: mTORC1 signaling, EMT, coagulation, Myc targets V1, protein secretion, xenobiotic metabolism, glycolysis, cholesterol homeostasis, apical junction, and complement. In the hormone-treated group, the pathways included mTORC1 signaling, coagulation, Myc targets V1, reactive oxygen species (ROS) pathway, protein secretion, apical junction, myogenesis, epithelial-mesenchymal transition, glycolysis, and cholesterol homeostasis.

The shared pathways identified the two groups included mTORC1 signaling, coagulation, Myc targets V1, protein secretion, epithelial-mesenchymal transition, glycolysis, and cholesterol homeostasis. These pathways may play crucial roles in the function of endometrial EVs. However, the detection of additional ROS and myogenesis pathways specifically in the hormone-treated group indicates that hormone treatment may influence cellular oxidative stress responses and myogenic differentiation.

mTORC1 signaling and EMT are essential for embryonic growth and intercellular interactions, and the increase in factors related to ROS and myogenesis may represent vital mechanisms for maintaining an optimal environment for embryo implantation within the uterus. Therefore, the regulatory mechanisms of endometrial EVs are pivotal in embryonic development and implantation processes (Table 3).

**Table 3:** Top 10 significant terms (based on *p*-values) in MSigDB Hallmark 2020

Group	Term	*p*-value	Overlapping proteins
Control	mTORC1 signaling	2.521e-08	G6PD, TFRC, PSMD13, PITPNB, IDH1, USO1, ARPC5L, SORD, CACYBP, DHFR, STIP1, PFKL, COPS5, PSMC4, BCAT1, YKT6, ATP6V1D
	Epithelial mesenchymal transition	2.521e-08	ITGB1, TPM4, LUM, SERPINE1, FN1, BGN, TNC, LAMC1, THBS1, COL1A1, LGALS1, DPYSL3, SPP1, CD59, ITGA5, VIM, PFN2
	Coagulation	4.809e-08	CFD, ANXA1, PEF1, SERPINE1, FN1, F2, CLU, C8A, THBS1, PROC, C8G, CAPN2, A2M, CTSB
	Myc targets V1	1.513e-07	EIF4A1, RANBP1, NPM1, SET, PCNA, PA2G4, UBE2L3, PRDX3, PSMD7, COPS5, PSMB3, PSMC4, SRSF3, VDAC1, ACP1, CCT4
	Protein secretion	3.194e-06	RAB2A, NAPA, SNX2, KRT18, CLTC, USO1, GNAS, SEC22B, YKT6, NAPG
	Xenobiotic metabolism	2.145e-05	CYFIP2, ITIH4, JUP, IDH1, SERPINE1, ADH5, CNDP2, VTN, VNN1, FETUB, BCAT1, ACP1, ALDH9A1
	Glycolysis	9.595e-05	GYS1, G6PD, GNPDA1, PSMC4, IDH1, PMM2, GFPT1, TALDO1, MIF, TXN, PGLS, ALDH9A1
	Cholesterol homeostasis	1.807e-04	PLSCR1, FASN, ANXA5, MAL2, CLU, GNAI1, S100A11
	Apical junction	3.943e-04	VASP, ITGB1, RRAS, JUP, ARPC2, MDK, SRC, ACTN1, MSN, PARVA, GNAI1
	Complement	3.943e-04	CPM, PLSCR1, PPP4C, SRC, SERPINE1, ANXA5, FN1, CD59, F2, CLU, CTSB
Hormone	mTORC1 signaling	3.092e-09	G6PD, TFRC, PSMD13, PITPNB, IDH1, USO1, ARPC5L, M6PR, SORD, CACYBP, DHFR, STIP1, PFKL, COPS5, PSMC4, EEF1E1, BCAT1, YKT6, ATP6V1D
	Coagulation	2.372e-08	CFD, SERPINA1, ANXA1, CFH, PEF1, APOC3, CRIP2, F2, CLU, C8A, PROC, C8G, CAPN2, A2M, CTSB
	Myc targets V1	1.088e-07	EIF4A1, RANBP1, SET, PCNA, PA2G4, UBE2L3, LSM2, PSMD7, COPS5, PSMB3, PSMC4, SRSF2, EIF4H, SRSF3, VDAC1, ACP1, CCT4
	Reactive oxygen species pathway	2.132e-06	PTPA, PRDX2, G6PD, GPX4, STK25, MGST1, TXN, FTL
	Protein secretion	7.777e-06	RAB2A, SNX2, KRT18, CLTC, USO1, M6PR, GNAS, SEC22B, YKT6, NAPG
	Apical junction	6.074e-05	VASP, ITGB1, JUP, SRC, ACTN1, MSN, PARVA, ACTN4, BAIAP2, GNAI1, RRAS, ARPC2, MDK
	Myogenesis	2.450e-04	EIF4A2, CFD, COL1A1, ITGB1, FABP3, SYNGR2, TPM3, COL6A3, MYH11, TPD52L1, CLU, CNN3
	Epithelial mesenchymal transition	2.450e-04	COL1A1, ITGB1, LGALS1, TPM4, LUM, ANPEP, DPYSL3, COL6A3, CD59, LAMC1, ITGA5, PFN2
	Glycolysis	2.450e-04	G6PD, GNPDA1, PSMC4, CASP6, IDH1, PMM2, GFPT1, CDK1, TALDO1, MIF, TXN, ALDH9A1
	Cholesterol homeostasis	3.338e-04	PLSCR1, FASN, ANXA5, MAL2, CLU, GNAI1, S100A11

### STRING and Reactome Analyses

A proteomic analysis of endometrial cell–derived EVs was conducted to evaluate differential expression between EVs derived from regular endometrial epithelial cells (control group) and EVs derived from hormone-treated endometrial epithelial cells (hormone group). Using STRING and Reactome for functional annotation, the top 10 Reactome pathways were identified for each group with an FDR threshold of <0.01.

In the control group, 276 DEPs were associated with pathways involved in the immune response, cell development, and cellular interactions. The prominent pathways identified included the formulation of the cornified envelope, innate immune system, and immune system, indicating roles in cellular defense and immune activation. Additional pathways, such as developmental biology, vesicle-mediated transport, and regulation of the complement cascade, further suggest contributions to both cellular formation and protective mechanisms. DEPs in the control group were involved in neutrophil degranulation, Fc-gamma receptor–dependent phagocytosis, and regulation of actin dynamics for phagocytic cup formation, highlighting their roles in phagocytic and immune-related cell functions. Lastly, cell–surface interactions at the vascular wall were observed, which may support cellular adhesion and communication during embryonic development.

**Fig. 5:**
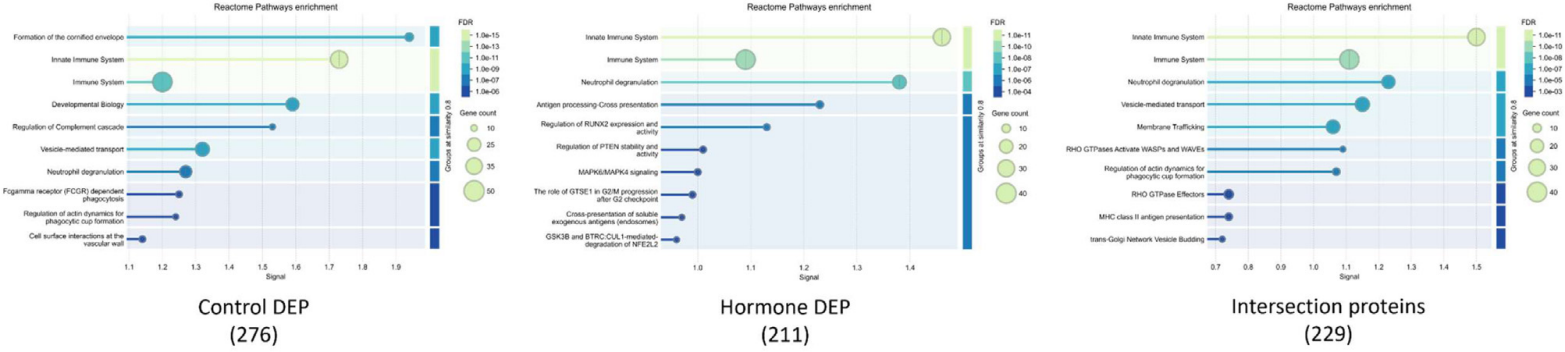
**Top enriched reactome pathways identified *via* STRING analysis in proteins from hormone groups and control groups**.

In the hormone group, 211 DEPs were associated with pathways involved in immune responses and specific cell signaling processes. Similar to the control group, pathways related to the innate immune system, immune system, and neutrophil degranulation were prominent, indicating that immune response pathways are a shared characteristic. Additionally, unique pathways, such as antigen processing with cross-presentation, regulation of RUNX2 expression and activity, and MAPK6/MAPK4 signaling, suggest that the EVs in the hormone group are involved in antigen presentation and regulatory signaling for cell differentiation. Other notable pathways included GTSE1 in G2/M progression after the G2 checkpoint, cross-presentation of soluble exogenous antigens in endosomes, and GSK3B and BTRC degradation of NFE2L2, which suggest functions in cell cycle regulation and protein degradation processes, which could influence cellular stability and growth during early embryonic development (Fig. 5).

## Discussion

In this study, we identified DEPs involved in distinct biological processes between control EVs and hormone-treated EVs, highlighting their roles in cellular mechanisms relevant to embryonic development. Proteins in the control group were involved in processes such as intermediate filament organization and cytoskeleton organization, which are crucial for maintaining cellular integrity and facilitating migration. These processes are known to support cell adhesion and migration, which are vital for successful implantation in the uterine environment. Since EVs serve as mediators of cell-to-cell communication, the identified proteins in pEEC-EVs may actively regulate embryo implantation by modulating cellular environments. For example, actin-binding proteins (ACTN4, CFL1) and tubulin (TUBB) could enhance cytoskeletal integrity, facilitating trophoblast motility and invasion (34). Likewise, molecular chaperones (HSPA8, HSP90B1) may support stress adaptation and protein homeostasis within the endometrial microenvironment (35, 36). These results suggest that pEEC-EVs contribute not only to the transport of bioactive molecules but also to the regulation of cellular dynamics essential for successful implantation.

This difference in biological functions suggests an adaptation to hormones, potentially impacting the efficiency of intercellular signaling and communication, which are critical during early embryogenesis.

Notably, vesicle-mediated transport and protein localization were key processes in the hormone group. These processes are essential for directing proteins to their functional sites within the cell, thereby ensuring proper cellular responses to developmental cues. The regulation of these pathways might influence the responses of embryos to the microenvironment, particularly in the context of implantation and early development (37, 38).

Furthermore, the regulation of the cellular response to stress was an important pathway in the hormone-treated group, potentially representing a mechanism for adaptation to environmental changes during early embryonic stages. The ability of embryos to respond to stress can impact their viability and developmental success, underscoring the significance of these biological processes in the context of implantation and overall embryonic health (39, 40). In summary, the findings from this study indicate that hormonal conditions influence the biological processes involved in embryonic development substantially. The specific processes emphasize the intricate relationship between cellular organization, protein transport, and the ability of embryos to thrive in their developmental environment.

An analysis of signaling pathways in pEEC-EVs revealed that EVs in both control and hormone-treated groups have important roles in embryonic development and implantation. The VEGFA VEGFR2 signaling pathway was a key pathway in both groups, particularly in the hormone-treated group, where it may enhance angiogenesis and vascular permeability. This modulation supports the supply of nutrients and oxygen to the embryo during early implantation, indicating that hormones optimize the endometrial environment to improve embryo viability (41, 42).

In the control group, focal adhesion and regulation of actin cytoskeleton pathways were identified, highlighting the role of these proteins in cell–matrix interactions and cellular dynamics necessary for embryo attachment to the uterine wall (43). These pathways are essential for implantation, even in the absence of hormonal stimuli (44). Among the proteins identified in the focal adhesion pathway, integrins (ITGAV, ITGB3) play a crucial role in mediating direct adhesion between the embryo and the endometrial lining (45, 46). Additionally, decreased expression of E-cadherin (CDH1) in response to TGF-β signaling promotes EMT, facilitating trophoblast invasion into the endometrium (47).

The identification of proteins involved in the IL-24 signaling pathway in the hormone-treated EVs suggests a role in immune modulation, potentially aiding in the establishment of an immune environment conducive to embryo acceptance (48, 49). The control group showed relatively less activation of immune-related pathways, emphasizing the potential for hormonal treatment to enhance immune responses that facilitate implantation. IL-24, identified in the hormone-treated group, has been linked to immune tolerance mechanisms, potentially reducing maternal immune rejection and facilitating embryo implantation. This suggests that pEEC-EVs may contribute to the establishment of an immune-permissive environment essential for implantation success.

Overall, these findings highlight the potential of pEEC-EVs in improving implantation success by modulating immune responses and cellular environments. Further studies should explore the molecular mechanisms underlying these effects for applications in ART.

In pEEC-EVs, the TGF-β signaling pathway identified in the control group plays a crucial role in enhancing cell motility and adhesion, facilitating the attachment of embryos to the uterine endometrium. Additionally, the VEGF pathway promotes angiogenesis, which is essential for early embryonic development by improving nutrient and oxygen supply. The focal adhesion pathway provides the fundamental structure necessary for embryo attachment to the uterine endometrium, which is critical for normal implantation (50, 51).

In the hormone-treated group, the proteins were predominantly involved in regulating immune responses and cellular homeostasis. Notably, the proteasome degradation pathway contributes to maintaining intracellular environments by removing abnormal proteins (52). The complement and coagulation cascades pathway provides signals that may influence immune responses and embryonic development (53, 54). Hormone-induced changes in EV cargo likely optimize the uterine environment for implantation (55). Enrichment of immune-regulatory proteins (IL-24, complement proteins) may enhance maternal tolerance, while stress-response proteins (HSPA8, HSP90B1) support embryo survival (4). These findings suggest that EV cargo modulations actively contribute to implantation success by balancing immune responses and cellular adaptation. Beyond implantation, EV-mediated signaling may influence subsequent embryonic development. EVs contribute to trophoblast differentiation and placental formation, which are essential for nutrient exchange and fetal growth (56). Additionally, EV cargo may regulate key developmental pathways, such as Wnt and mTOR signaling, impacting organogenesis and fetal programming (57). Future research should explore the long-term effects of EVs on postimplantation development and pregnancy outcomes.

Broadly, these results contribute to a deeper understanding of the role of pEEC-EVs in embryonic development and implantation. Future research should aim to elucidate the specific mechanisms of action of each pathway and protein as well as potential clinical applications.

A gene set enrichment analysis of pEEC-EVs indicated that hormone treatment may significantly influence embryonic development and implantation processes. The pathways identified in the control group, including mTORC1 signaling, EMT, and protein secretion, are critical for embryonic growth and intercellular interactions (50, 51). The additional identification of the ROS pathway and myogenesis in the hormone-treated group reflects the potential effects of hormones on oxidative stress responses and differentiation processes.

These results imply that hormone-treated EVs may modulate the cellular microenvironment, thereby facilitating optimal implantation conditions for the embryo (58). Future studies should determine the specific roles of these pathways and the functional characteristics of endometrial EVs. Such investigations could enhance our understanding of the roles of EVs in embryonic development and implantation, ultimately contributing to the development of therapeutic strategies for pregnancy-related disorders.

The differential proteomic analysis suggested that EVs derived from regular endometrial epithelial cells and EVs derived from hormone-treated endometrial epithelial cells have distinct roles in supporting porcine embryonic development. These findings suggest that EVs may be used to optimize *in vitro* embryo culture conditions, enhancing embryo development and implantation potential in ART settings. Developing EV-based culture supplements or delivery systems could further improve embryo quality and implantation outcomes. The control EVs were primarily involved in pathways related to structural and immune functions, such as formulation of the cornified envelope, vesicle-mediated transport, neutrophil degranulation, and regulation of the complement cascade. These pathways imply that control EVs play essential roles in early cell interactions, immune protection, and structural stability within developing embryos (59). In contrast, pathways activated in EVs derived from hormone-treated cells were prominently associated with regulatory signaling, including MAPK6/MAPK4 signaling and RUNX2 expression regulation, as well as immune functions, suggesting potential contributions to cell differentiation and cell cycle regulation, essential for embryonic development. Both groups demonstrated the activation of innate immune responses, highlighting the crucial role of endometrial-derived EVs in immune support during early embryonic stages. Overall, these findings indicate that while control EVs may primarily promote foundational structural and immune processes, hormone-treated EVs could uniquely promote cell growth and differentiation, thereby contributing to the developmental microenvironment of porcine embryos (60).

These findings align with previous reports in human and bovine models, where VEGFA-VEGFR2 signaling has been shown to enhance vascular remodeling at the implantation site (61). Additionally, the observed immune-modulatory effects of pEEC-EVs support the hypothesis that EVs contribute to implantation success by regulating maternal immune tolerance, consistent with findings in murine models (62).

In conclusion, this study highlights significant differences in biological processes and signaling pathways between control and hormone-treated pEEC-EVs. Hormones alter cellular organization, protein transport, and immunity, thereby improving conditions for embryo implantation and development. The pathways identified in this study underscore the intricate relationship between hormonal signaling and the microenvironment necessary for successful embryogenesis. Future research should further explore these pathways to develop targeted therapeutic strategies for improving reproductive health.

## Data availability

All data used in this study are publicly available. Proteomic data are available from PRIDE (Project accession: PXD060211).

## Supplemental data

This article contains supplemental data.

## Conflicts of interest

The authors declare no competing interests.

## SupplementaryData

Supplementary results of mascot search
